# Beyond the Classic: Atypical Dengue Presentations

**DOI:** 10.7759/cureus.104973

**Published:** 2026-03-10

**Authors:** Nandu M, Kathyayini Revivarman, Manna M Theresa

**Affiliations:** 1 Emergency Medicine, Dr Somervell Memorial CSI Medical College, Thiruvananthapuram, IND; 2 Emergency Medicine, Government Medical College, Thiruvananthapuram, Thiruvananthapuram, IND; 3 Emergency Medicine, Amrita Institute of Medical Sciences, Kochi, IND; 4 Emergency Medicine, Amala Institute of Medical Sciences, Thrissur, IND

**Keywords:** dengue, dengue expanded syndrome, encephalitis, hemophagocytic lymphohistiocytosis, syndrome of inappropriate secretion of antidiuretic hormone (siadh)

## Abstract

Dengue is an arthropod-borne viral infection caused by the dengue virus and spread by the vector Aedes mosquitoes. The clinical spectrum of dengue includes undifferentiated fever, dengue fever, dengue hemorrhagic fever, dengue shock syndrome, and expanded dengue syndrome (EDS). The patients may present with atypical symptoms and involve organs like the liver, kidney, brain, heart, bone marrow, and central nervous system. Three atypical dengue cases belonging to the EDS that had presented to the Emergency Department of a tertiary care center in Kerala, South India, are described in the case series. The first patient developed hemophagocytic lymphohistiocytosis secondary to dengue infection. The second patient had presented with altered mental status and was diagnosed with dengue encephalitis. The third patient had hyponatremia-syndrome of inappropriate anti-diuretic hormone secretion along with dengue infection. With a rapid increase in dengue cases worldwide, atypical presentations of dengue are no longer uncommon. Timely recognition and prompt management of the atypical presentation are needed, as many of the manifestations have serious outcomes. Hence, clinicians should be aware of the atypical manifestations of dengue.

## Introduction

Dengue is an arthropod-borne viral infection caused by dengue virus (DENV), belonging to the Flaviviridae family, which has four subtypes: DENV-1, DENV-2, DENV-3, and DENV-4. The Aedes mosquitoes, Aedes aegypti and Aedes albopictus, are common vectors [1,2]. The clinical spectrum of dengue includes undifferentiated fever, dengue fever, dengue hemorrhagic fever (DHF), dengue shock syndrome (DSS), and expanded dengue syndrome (EDS) [2]. Dengue infections will mostly be asymptomatic or will present with mild symptoms. Dengue fever mostly presents as an acute febrile illness with symptoms like headache, retro-orbital pain, arthralgia, myalgia, and rashes along with thrombocytopenia and leukopenia [2]. DHF presents with features of dengue fever along with bleeding manifestations like petechiae, ecchymoses, mucosal bleeding, and gastrointestinal bleeding in severe cases. DSS is characterized by hypovolemic shock secondary to increased capillary permeability and plasma leakage [2]. The dengue infections that could not be described by the criteria for DHF or DSS, presenting with severe organ dysfunction, were termed EDS. Patients may present with atypical symptoms and involve multiple organs. These atypical presentations may be the result of severe shock, pre-existing diseases in the patient, or co- infections [2,3-5]. Though dengue incidence has shown a decrease during the initial phase of the COVID-19 pandemic, there has been a drastic rise in dengue cases both globally and in India [1]. Three atypical cases of dengue or EDS that were presented to the Emergency Department of a tertiary care center in Kerala, South India are described in this case series. Hemophagocytic lymphohistiocytosis (HLH), encephalitis, and syndrome of inappropriate anti-diuretic hormone secretion (SIADH) are rare presentations in dengue. Awareness of these atypical presentations associated with dengue helps in early diagnosis and timely management.

## Case presentation

Case 1: dengue with secondary hemophagocytic lymphohistiocytosis

**Figure 1:**
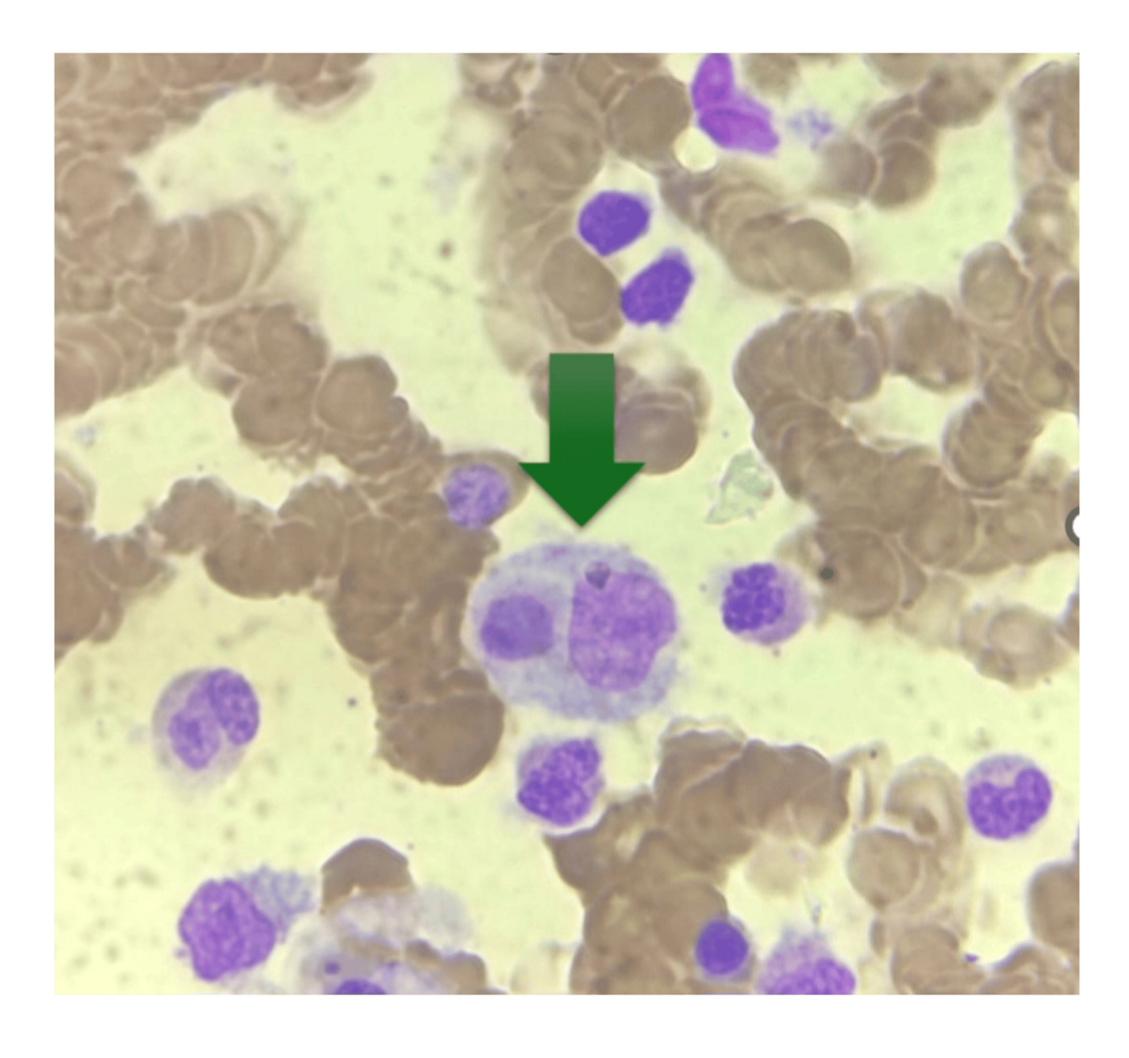
Bone marrow aspiration study showing normocellular marrow with erythroid hyperplasia and features of hemophagocytosis.

A 23-year-old male migrant worker with no co-morbidities and addictions presented to the emergency room (ER) with complaints of fever with chills and myalgia for four days. He had associated headaches and giddiness. The patient’s vital signs were stable, heart rate (HR) of 86 beats per minute (bpm), blood pressure (BP) of 120/80mm of Hg, SpO2 of 98% in room air, and respiratory rate (RR) of 22/minute. On systemic examination, the abdomen was soft and had a palpable spleen. Other system examinations were within normal limits. Patient’s blood investigations revealed pancytopenia (total white blood cell (WBC) count: 1600 cells/cu mm, hemoglobin (Hb): 6.6mg/dL, and platelet count: 28000/cu mm), indirect hyperbilirubinemia with elevation of liver enzymes (total bilirubin: 2.2mg/dL, conjugated bilirubin: 0.4mg/dL, SGOT: 622 units/L, and SGPT: 332 units/L), hyperferritinemia (4021ng/mL), and elevated inflammatory markers (LDH: 571U/L and CRP: 25.4mg/L). Serological tests to rule out tropical fevers (dengue, malaria, leptospirosis, scrub typhus) and HIV were done. The dengue NS1 antigen test was positive. Peripheral blood smear showed pancytopenia with no atypical cells, blast cells, or parasites. The blood culture was sterile. Ultrasonography of the abdomen showed splenomegaly. The patient was treated with piperacillin-tazobactam and fluconazole and was transfused with one unit of whole blood. Fluid management based on inferior vena cava (IVC) collapsibility, along with other symptomatic and supportive treatment, was provided. However, the patient had further worsening of pancytopenia (platelet: 13000 cells/cu mm, WBC: 1020cells/uL, Hb: 7mg/dL) along with uptrending ferritin levels (24880ng/mL). The patient also had a persistent fever. Further testing revealed hypertriglyceridemia (103mg/dL) and hypofibrinogenemia (180mg/dL). The patient was transfused with four units of random donor platelets, and a bone marrow aspiration study was done to confirm the diagnosis of HLH. It showed normocellular marrow with erythroid hyperplasia and evidence of hemophagocytosis (Figure 1).

The diagnosis of HLH was made based on HLH 2004 criteria, a HScore of 224 points (suggestive of 96-98% probability of hemophagocytic syndrome), and the bone marrow aspiration study. The patient was started on intravenous dexamethasone pulse therapy for three days, followed by a tapering dose of oral dexamethasone. The patient had shown improvement clinically, and the laboratory parameters also improved within a span of 48 hours of initiating steroid therapy (Table 1). After seven days of hospital stay, the patient was discharged home, and the lab parameters were followed up on.

**Table 1:** Trends in laboratory investigations over the days of hospital admission.

	Day 1	Day 2	Day 4	Day 7	Day 10
Hemoglobin(g/dL)	6.6	7	8.1	8.8	9
Total leucocyte count (cells/cu mm)	1600	1020	1800	4300	6100
Platelet count (/cu mm))	28000	13000	42000	96000	114000
SGOT (units/L)	622	840	512	119	86
SGPT (units/L)	332	412	308	78	60
Ferritin (ng/mL)	4021	24880	7341	2104	364
LDH (U/L)	571	1108	889	212	158
Triglycerides (mg/dL)	Not done	103	92	87	72
Fibrinogen (mg/dL)	Not done	180	110	78	58

Case 2: dengue encephalitis

**Figure 2:**
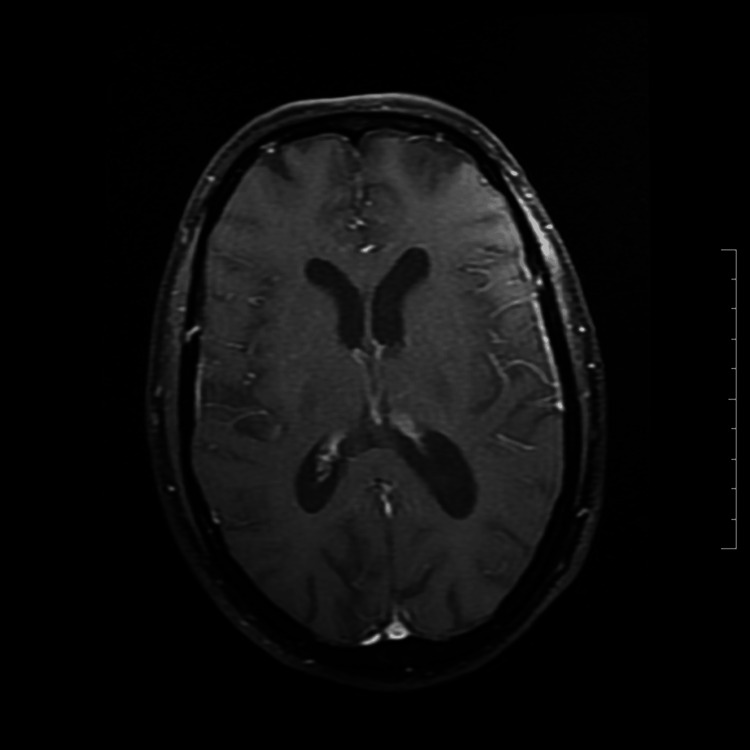
MRI brain with contrast showing irregular non-continuous leptomeningeal enhancement.

A 70-year-old man with type 2 diabetes mellitus, dyslipidemia, and systemic hypertension with a history of recent COVID-19 infection presented to the ER with complaints of a few episodes of vomiting, unsteadiness of gait, and slurred speech for two days. On initial assessment, the patient was febrile (temperature: 100.4F) and tachycardic (HR: 107bpm). The patient was conscious and oriented to place, person, and time. The patient had normal tone and power in all limbs. The straight-line walking test and finger-nose test were positive. The patient’s initial blood tests were normal except for mild thrombocytopenia (platelet count: 1,45,000cells/cumm). MRI brain (DWI, ADC, and T2FLAIR) was done and was normal. Dengue NS1 and IgM were done in view of fever with thrombocytopenia, and dengue IgM was positive. The patient was managed symptomatically; the platelet count was monitored and was stable. However, the patient had persistent fever and headache. On the second day of hospitalization, the patient developed altered sensorium. The patient’s blood sugars, electrolytes, and blood counts were within normal limits. Fundoscopic evaluation revealed signs of early papilledema. MRI brain with contrast was done, which showed irregular non-continuous leptomeningeal enhancement (Figure 2).

The patient’s CSF study showed 75 cells (85% mononuclear; normal range: 0 to 5 cells), protein (119.6 mg/dL; normal range: 15 to 40 mg/dL), and glucose (78.4mg/dL; normal range: 50 to 80mg/dL), suggestive of viral meningitis. CSF gram staining was also negative. The patient was started on intravenous ceftriaxone, acyclovir, and steroids. In view of the dengue infection, CSF was also tested, and dengue IgG and IgM antibodies were detected. The patient had a worsening of sensorium and was hence mechanically ventilated. Other viral causes of encephalitis were ruled out by CSF PCR tests. Both bacterial and fungal cultures of CSF were negative. Autoimmune and paraneoplastic panel tests were also negative in CSF. The patient continued with the same treatment for two weeks. The patient had a gradual improvement, was weaned off the ventilator in a week, and reached baseline sensorium over three weeks. The patient could walk with support and was discharged from the hospital.

Case 3: dengue with SIADH

A 68-year-old woman with systemic hypertension and dyslipidemia presented to the ER with complaints of fever with chills and headache for three days. The patient also had complaints of nausea and vomiting. The patient’s initial vital signs were stable, and systemic examination didn’t reveal any abnormality. Patient’s lab tests revealed thrombocytopenia (80000 cells/cumm), elevated inflammatory markers (CRP: 25.4 mg/dL), and hyponatremia (124 mmol/L). A fever workup was done (dengue NS1, peripheral smear for plasmodium parasite, IgM Leptospirosis, and IgM Scrub Typhus). Dengue NS1 was positive. The COVID antigen test, other serological tests, and peripheral smear for parasites were negative. Lab tests revealed high urine sodium (104.7 mmol/L) and urine osmolality (412 mOsm/kg) with a low plasma osmolality (259mOsm/kg). The thyroid function test and serum cortisol were normal. The patient had no signs of intravascular fluid depletion or excess, as confirmed with IVC diameter and collapsibility in point-of-care ultrasound. A diagnosis of SIADH was made, and the patient was advised fluid restriction to maintain a negative balance and also high salt intake. The patient was managed conservatively and symptomatically. The patient’s platelet and sodium levels were monitored and gradually improved. The patient was discharged after five days of hospital stay (Table 2).

**Table 2:** Trends of laboratory investigation over the period of hospital admission

	At Admission	At Discharge
Hemoglobin (g/dL)	12.5	12.4
Total leucocyte count (cells/cu mm)	4800	5400
Platelet count (/cu mm))	80000	120000
SGOT (units/L)	49.9	32
SGPT (units/L)	18.8	15.4
Ferritin (ng/mL)	208	
LDH (U/L)	299	
Creatinine (mg/dL)	0.86	0.78
Sodium (mmol/L)	124	136
Potassium (mmol/L)	3.6	3.8
Serum osmolality (mOsm/kg)	259	
Urine osmolality(mOsm/kg)	412	
Urine sodium (mmol/L)	104.7	

The three cases discussed above have been included after obtaining written informed consent from the patients' legal guardians.

## Discussion

Dengue infections have a global case burden of millions, particularly in the tropics and sub-tropics [1,2]. The World Health Organization (WHO) in 1997 described three categories of dengue infections based on symptoms, i.e., dengue fever, DHF, and DSS. A revised classification was described by the WHO in 2009, classifying dengue into dengue without warning signs, dengue with warning signs, and severe dengue. The 2011 WHO guidelines brought up the novel concept of EDS, in which patients develop unusual presentations involving different organs with significant severity. EDS cases are those that do not fall into the category of either DHF or DSS [2,3].

The pathogenesis of EDS is due to different mechanisms, like the neutropenic effect of viruses, direct invasion by viruses, immune-mediated injury, hypoxic injury, plasma leakage syndrome, and coinfections [6]. Clinical presentations of EDS involving the gastrointestinal and hepatic systems include transaminitis, fulminant hepatic failure, acalculous cholecystitis, pancreatitis, and intestinal obstruction. Cardiovascular involvement manifests as myocarditis, heart blocks, pericarditis, cardiomyopathy, and arrhythmias like atrial fibrillation. EDS can present with neurological manifestations like encephalitis, encephalopathy, meningitis, Guillain-Barré syndrome, peripheral mono or poly neuropathy, and transverse myelitis. Renal presentations include acute kidney injury, nephritic syndrome, glomerulonephritis, and hemolytic uremic syndrome. Electrolyte abnormalities like hypokalemia, hyponatremia, and SIADH are also reported. Coinfections of malaria, chikungunya, leptospirosis, Zika Virus, and COVID-19 with dengue have also been known [3-6].

HLH secondary to dengue fever occurs as a response to dysregulated macrophage activation, causing cytokine storm and immune-mediated tissue destruction [7]. In addition to the features like fever, thrombocytopenia, anemia, elevated ferritin levels, and liver dysfunction seen in severe dengue, there are specific features like splenomegaly, elevated LDH, elevated triglyceride levels, low fibrinogen levels, and bone marrow features of hemophagocytes in HLH [8,9]. Patients with HLH secondary to dengue mostly have features of severe dengue. Hence, there is a chance the clinician could overlook the diagnosis of HLH. Diagnosis can be made based on HLH 2004 or modified HLH 2009 criteria [8,9]. A short course of intravenous steroids, methylprednisolone or dexamethasone, is known to produce better clinical outcomes [9,10]. In a study by Kan et al., eight out of 10 patients with HLH treated with steroids alone had survived [10]. Similarly, Ponnuraj et al. have reported the case of an eleven-month-old baby showing a good outcome with dexamethasone, as experienced with our case [7]. Intravenous immunoglobulin alone or with steroids and Etoposide has also been used in some case scenarios [8,9,11]. Though dengue-induced HLH is a rare entity, it poses a high mortality risk, and hence early detection and management are significant [11,12].

Dengue infection can present with various atypical neurological manifestations like encephalitis, encephalopathy, meningitis, seizures, Guillain-Barré Syndrome, peripheral mono or poly neuropathy, and transverse myelitis [4,13]. Neurological manifestations can occur in dengue due to (i) DHF, leading to fluid leak and hyponatremia, resulting in cerebral edema, (ii) Neurotropic action of viruses, (iii) Auto-immune and immune-mediated effects, and (iv) Metabolic derangements [13]. Dengue encephalitis occurs as a result of inflammation of the brain parenchyma due to the neurotropic effect of the dengue virus. Fever associated with features of cerebral parenchyma involvement like seizures, altered mental status, and/or focal deficits, IgM dengue antibody or NS1 antigen or dengue PCR positivity in CSF and/or serum, and absence of other causes of viral encephalitis form diagnostic features of dengue encephalitis [14]. MRI of brain with T2 sequences shows hyperintensities that help in diagnosing dengue encephalitis [13]. Symptomatic and supportive management, like adequate hydration, antiepileptic drugs to control seizures, and anti-edema measures like mannitol and steroids, are used to manage dengue encephalitis [15]. Dengue encephalitis has a good recovery rate, but a mortality rate of 3.4% was reported in a study by Jackson et al. [16]. In a study by Solomon et al. done in Vietnam, no patients with dengue encephalitis in their study group had died, but two-thirds of them had neurological sequelae [17]. In our case, too, the patient recovered with minimal residual neurological deficits. In patients presenting with encephalitis, dengue should be among the differential diagnosis at least in dengue endemic regions. 

Hyponatremia is a common electrolyte abnormality in dengue infections. Hyponatremia can occur in dengue fever due to SIADH, reduced renal excretion, increased water from increased metabolism, and sodium potassium pump dysfunction, causing sodium influx into cells. To confirm the diagnosis of SIADH, the patient should have low serum osmolality (less than 275 mOsm/kg), high urine sodium concentration (more than 40 mEq/L), with normal thyroid and adrenal functions, with normal levels of blood glucose, triglycerides, and proteins, and with no signs of extracellular fluid volume excess or depletion [18,19]. In our case, the diagnosis of SIADH was made after confirming all these diagnostic criteria. In the treatment of SIADH, excess salt is provided and is not lost through other sources. The patient’s intravascular volume is maintained. To create a negative balance and to bring osmolality back to normal, fluid restriction is done [18,19]. Management of hyponatremia should be tailored to the fluid status. In the presence of capillary leakage in DHF, fluid restriction will worsen intravascular hypovolemia. The case of a 40-year-old male with dengue-induced SIADH reported from Bangladesh was treated with 3% hypertonic saline and V2 vasopressin antagonist (Tolvaptan) instead of fluid restriction to maintain perfusion pressure and to prevent further hyponatremia-induced cerebral edema [18]. SIADH in dengue is a rare occurrence, and prompt management helps to correct the hyponatremia without proceeding to complications. 

## Conclusions

Dengue infection has a wide spectrum of presentations, asymptomatic dengue, DHF, DSS, and atypical manifestations or EDS. Against the background of an increasing number of dengue cases globally and also in different parts of India, the atypical manifestations are no longer uncommon. Since many of the manifestations have serious outcomes, timely recognition and prompt management of the atypical presentation are needed. Hence, clinicians should be aware of the atypical manifestations of dengue, especially in endemic areas, to ensure prompt and timely management.
